# From a word to a world: the current situation in the interdisciplinary field of synthetic biology

**DOI:** 10.7717/peerj.728

**Published:** 2015-01-22

**Authors:** Xiaojun Hu, Ronald Rousseau

**Affiliations:** 1 Medical Information Centre, Zhejiang University School of Medicine, Hangzhou, China; 2 Institute for Education and Information Sciences, IBW, University of Antwerp (UA), Antwerp, Belgium; 3 Department of Mathematics, KU Leuven, Leuven, Belgium

**Keywords:** Query formulation, Synthetic biology, Year-based h-indices, Keyword analysis

## Abstract

Using a carefully designed search query, we describe the field of synthetic biology in terms of leading countries, organizations and funding sources. Besides articles we also paid some attention to patents. The USA is the leading country in this field, followed by China. There is a clear exponential growth in the field of synthetic biology over the latest 14 years. Keywords were analyzed using the notion of year-based h-indices, core gap and relative core gap. We conclude that the term “synthetic biology” hides a large world ready to be explored by interdisciplinary research.

## Introduction

Synthetic biology can be defined as the application of engineering principles to the fundamental components of biology. More precisely The Royal Society (2014) describes synthetic biology as follows:

*Synthetic biology is an emerging area of research that can broadly be described as the design and construction of novel artificial biological pathways, organisms or devices, or the redesign of existing natural biological systems.*

The European NEST High-Level Expert Group (2005) defines synthetic biology as follows:

*“Synthetic biology is the engineering of biology: the synthesis of complex, biologically based (or inspired) systems which display functions that do not exist in nature. This engineering perspective may be applied at all levels of the hierarchy of biological structures—from individual molecules to whole cells, tissues and organisms. In essence, synthetic biology will enable the design of ‘biological systems’ in a rational and systematic way.”*

The title of this article is derived from De Lorenzo & Danchin (2008) who described the current state at that time.

The purpose of this investigation in descriptive informetrics is to explain the current situation of this emerging field. In order to extract the necessary information, we performed a topic mining exercise in the Web of Science (WoS). The main step in this exercise is the construction of a search query in order to catch the essential components of the field. Our query is more comprehensive and leads to better results than that used by Oldham, Hall & Burton (2012), described in the next section. The results of this query enable the detection of the most active countries/regions, continents and organizations. In order to broaden the set of retrieved articles, we performed an additional search in PubMed and MEDLINE.

Not surprisingly, the USA is the most active country while Mainland China is moving up the ranks to become second in the last year. We further determine the WoS categories and areas to which articles on synthetic biology belong. It is shown that the growth in terms of number of published articles per year follows an exponential curve. These aspects are of interest and form an essential part of the study of any emerging field, but they do not use any new technique. Yet for the study of the distribution of topic keywords we apply a recently introduced approach (Hu & Rousseau, 2014a) based on the idea of year-based h-indices (Mahbuba & Rousseau, 2013). Details of the method are provided in the ‘Methods’ section. It is found that protein engineering, metabolic engineering and protein design are the overall hot topics in synthetic biology.

This article is an extended and reworked version of a preprint deposited in the arXiv (Hu & Rousseau, 2014b).

## A Short History and Review of the Field

The term “synthetic biology” was first introduced by the French scientist Stéphane Leduc (1912) although with a different meaning than today’s version, and according to Wikipedia in modern times by the Polish geneticist Waclaw Szybalski (Szybalski, 1974). Although Wikipedia provides a quote, we were unable to find this quote in Szybalski (1974). To be precise, Szybalski describes what we would nowadays call synthetic biology and writes: “we enter the synthetic phase of research in the field” (i.e., of molecular biology). Putting aside the question of who was first, it is true that the term gained popularity and usage in mainstream science only in the year 2004 when the first international meeting, called Synthetic Biology 1.0, was held at the Massachusetts Institute of Technology (Jain, Bhatia & Chugh, 2012). Going back to Fleming’s discovery of penicillin (the first antibiotic with wide-spread use) Jain and her collaborators discuss the scope of synthetic biology for developing novel drugs. Envisaging many other applications, scientists nowadays declare that they can do better than evolution (Schuster, 2013). Schuster points to promising aspects for information storage, recalling a pilot study (Church, Gao & Kosuri, 2012) in which an entire book, including figures and Javascript, totalling more than five megabits, were stored on a single DNA molecule. Goldman (2014) points out that the near-completion of the Human Genome Project provided the impetus for significant disciplinary progress. Reviews on synthetic biology, from a field-specialist technical point of view, can be found in Li & Vederas (2009), Purnick & Weiss (2009) and Esvelt & Wang (2013). Moreover, Purnick & Weiss (2009) as well as Esvelt & Wang (2013) provide a timeline of milestones in synthetic biology.

The main article to use informetric techniques to study the field of synthetic biology is by Oldham, Hall & Burton (2012). They explore the field to inform debates on the governance (related to the United Nations Convention on Biological Diversity) of the field. For this reason they focus on different visualizations. Based on WoS data they distinguish between two groups of articles: the core consisting of 1,255 publications and a group of articles citing the core leading to another 5,995 items. Searches were conducted in January 2012. Their core was obtained by a topic search for “synthetic biology,” “synthetic genomics,” “synthetic genome” or “synthetic genomes.” Details are discussed later in this article when comparing their results with ours. We note that Oldham, Hall & Burton (2012) observed the incipient diversification of synthetic biology into mammalian synthetic biology, cell free synthetic biology, chemical synthetic biology, genome engineering, genome-scale synthetic biology, and even more. They point out that this diversification is important for policy debates, as synthetic biology may cease to be a ‘unitary’ object for policy action.

Recently Goldman (2014) studied the related field of systems biology, using it as an empirical example to explore changes in the disciplinary structure of a field. She works under the assumption that concepts from systems biology are transmitted by papers linked via journals to various disciplines (in practice WoS subject categories). Following Liu & Rousseau (2010) she notes that the subject categories of the journals publishing on a topic can be indicators of the breadth of disciplinary diffusion. The author used a bipartite network to explore connectivity and concretely betweenness centrality among subject categories and journals. From 2000–2011 the number of subject categories and the number of journals both increased, while the percentage of subject categories with betweenness centrality equal to zero decreased. Such a decrease did not occur for the percentage of journals with betweenness centrality equal to zero. By 2011 subject categories formed a single large component. The whole structure can be characterized as a core/semi-periphery/periphery structure. Biotechnology & Applied Microbiology, Biochemistry & Molecular Biology, Computer Science, Mathematical & Computational Biology, Biophysics, and Genetic & Heredity form the core. Pharmacology & Pharmacy, Toxicology, Immunology, Nutrition & Dietetics, and Neurosciences & Neurology are examples of intermediary fields belonging to the semi-periphery. She notes that growth at the periphery occurs largely through interdisciplinary journals. Goldman (2014) performed a study which revealed that, over time, several clinical disciplines move toward the core. Immunology, healthcare sciences & services and oncology are examples of such categories. This movement illustrates the progress made by systems biology in translating theory to practice. As a specific example of the efforts to bridge theory and practice, she mentions the creation of the human diseasome linking genotype and phenotype (Goh et al., 2007). Finally, she proposes a typology of journal roles in core bridges, intermediary bridges and reinforcers.

## Methods

### Construction of a search query

As the retrieval language for the WoS is a keyword language and not based on a controlled vocabulary or a thesaurus, we have to construct a specific search query similar to what has been done for the field of nanotechnology (Kostoff et al., 2006).

**Figure 1:**
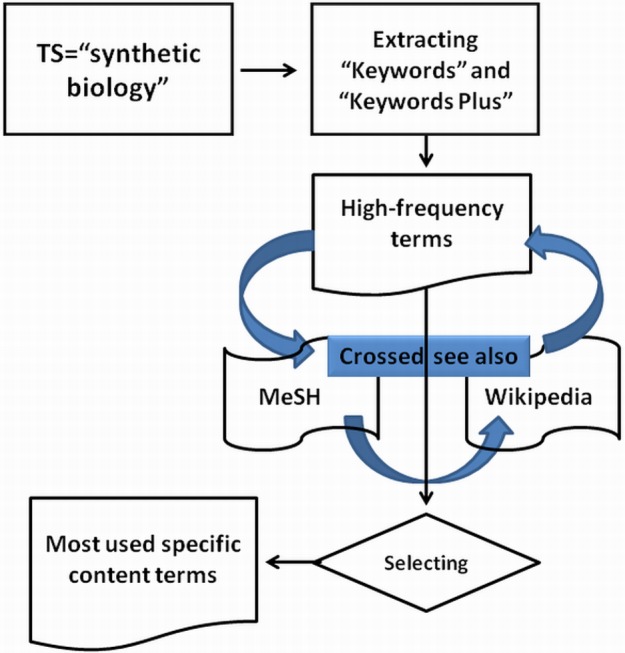
Search strategy for finding articles about synthetic biology.

The following methodology (synthesized in Fig. 1) has been employed.

(1) Essential records were retrieved using the term “synthetic biology” as a topic search in the Web of Science (in short: WoS):
  TS = “synthetic biology” and document type = article
  Databases = SCI-EXPANDED, SSCI, A&HCI, CPCI-S, CPCI-SSH Timespan = 2000–2013.
  This search retrieved 1,333 records (date of retrieval: January 11, 2014). These were downloaded using the full record option.
(2) Next, we extracted the “Keywords” and “Keywords Plus” from all 1,333 records, and obtained their frequencies. In this way 6,054 terms were found and ranked in descending order of occurrence.
(3) We sought the precise meanings of the terms in this list making use of MeSH (Medical Subject Headings) definitions and descriptions in Wikipedia (http://en.wikipedia.org/).
  This led to a list of most-used content terms related specifically to synthetic biology. The resulting list has been verified by a field expert.
(4) We then used these content terms related to synthetic biology to expand the original query, leading to the final search string:
  TS = (“synthetic biology” OR “synthetic gene network*” OR biobrick* OR “protein design*” OR “genetic circuit*” OR “gene regulatory network*” OR “cell-free protein synthes*” OR “metabolic engineering” OR “protein engineering” OR “promoter engineering” OR “DNA assembly” OR “RNA engineering biosensors” OR “multipart DNA assembly” OR “sequential circuits” OR “benchmark synthetic circuits” OR “DNA nanotechnology” OR “human artificial chromosome” OR “synthetic promoters” OR “transcriptional circuits” OR “abstract genetic regulatory network*” OR “gene assembly” OR “post-transcriptional regulation” OR “engineered proteins” OR “cell-free gene circuits”) AND Document Types: (Article).
  Databases = SCI-EXPANDED, SSCI, A&HCI, CPCI-S, CPCI-SSH
  Timespan = 2000–2013.

In this way, 13,836 records were obtained. This set is the main focus of this article. In order to broaden the view on the field of synthetic biology we also performed three other searches (in December 2014), starting with a search in PubMed with “synthetic biology” as a MeSH term. However, since this term was only introduced since the year 2011, this search only led to a small set of 821 journal articles. For this reason, we used the search string constructed for the WoS in the “advance search” of PubMed (http://www.ncbi.nlm.nih.gov/pubmed/advanced). This led to a search in the title/abstract field. In this way, we retrieved 12,028 journal articles published during the period 2000–2013. Finally, we queried MEDLINE via the Web of Knowledge (WoK) (https://apps.webofknowledge.com/MEDLINE_GeneralSearch). This approach has a better search interface and provides a search field for “topic search.” Using the same search string and restricting to the article type, we retrieved 27,208 journal articles published in the time span 2000–2013. All records were downloaded for further analysis. Table 1 synthesizes our search results.

**Table 1:** Search results by different interfaces.

	Search query	Time span	Records
PubMed/MeSH	Synthetic biology as MeSH term	2011–	821
PubMed/advanced	Search string applied to title/abstract	2000–2013	12,028
MEDLINE/WoK	Search string as a topic search	2000–2013	27,208
WoS	Search string as a topic search	2000–2013	13,836

We note that Goldman (2014) only used the topic search term “systems biology” in the WoS and included all publication types, leading to 4,446 publications over the period 2000 through 2011. This is a major difference between our approach and that of Goldman.

As the field of synthetic biology is said to hold great promise for commercialization, we also performed a search for patents in the Derwent Innovations Index (DII) using a similar search query as in the WoS. The search was performed on January 24, 2014 in CDerwent, EDerwent and MDerwent, and the timespan = 2000–2013. This resulted in 788 patent records.

### Data processing

- Topic keyword counting. We determined the keyword frequency based on the retrieved 13,836 records from the WoS search and their yearly distribution.
- Dynamic study of keyword use. To find out the major keywords in this field and their changes in frequency over the period 2000–2013, we calculate the value for the highly frequent keywords using the recently introduced “year based h-type indices” (Mahbuba & Rousseau, 2013; Hu & Rousseau, 2014a; Hu & Rousseau, 2014b).

## Results and basic data

In this section we show basic results: most active countries/regions, continents and organizations; WoS categories and areas to which articles on synthetic biology belong; number of articles per year and aspects of growth. Most data were obtained by using the WOS *analyze* function. Table 2 shows a list of most-cited articles in the field of synthetic biology according to the WoS.

**Table 2:** Most cited articles in the domain of synthetic biology (WoS results).

Authors	Source	Publication year	Times cited
Baba, T, et al.	Molecular Systems Biology, 2, article number 2006.0008	2006	1,654
Gardner, TS, et al.	Nature, 403(6767), 339–342	2000	1,379
Ren, B, et al.	Science, 290(5500), 2306–2309	2000	1,197
Mattoussi, H, et al.	Journal of the American Chemical Society, 122(49), 12,142–12,150	2000	1,134
Xie, XH, et al.	Nature, 434(7031), 338–345	2005	1,065
Zheng, M, et al.	Science, 302(5650), 1,545–1,548	2003	953
Chen, JF, et al.	Nature Genetics, 38(2), 228–233	2006	855
Kuhlman, B, et al.	Science, 302(5649), 1,364–1,368	2003	627
Canutescu, AA, et al.	Protein Science, 12(9), 2,001–2,014	2003	618
Wei, CL, et al.	Cell, 124(1), 207–219	2006	585

We abbreviated the term Synthetic Biology, referring to the set of articles retrieved by our WoS query as SB.

The most-active countries/regions over the period 2000–2013 are shown in Table 3. We added the leading country in Africa (South Africa) and Bangladesh as an example of a developing country and because of previous interest in it (Mahbuba & Rousseau, 2010). The WOS assigns an article to each country with at least one participating author as shown by his/her institutional address. In addition to rankings over the whole period, we also showed the number of publications and rankings for the first and the second half of the period. Moreover, we calculated the percentage of articles about synthetic biology among all articles (over the same period) and the ranking (restricted to the 27 countries/regions studied here) according to this parameter.

**Table 3:** Most-active countries/regions over the period 2000–2013: data from the WoS.

Rank	Country	# Articles	% SB and ranking	# Publications and ranking 2000–2007	# Publications and ranking 2008–2013
1	USA	5,973	0.144 (2)	2,419 (1)	3,554 (1)
2	Germany	1,392	0.129 (5)	504 (3)	888 (3)
3	Japan	1,294	0.125 (7)	630 (2)	664 (4)
4	People’s Republic of China	1,258	0.092 (16)	263 (5)	995 (2)
5	England	966	0.101 (13)	347 (4)	619 (5)
6	France	621	0.080 (19)	244 (6)	377 (6)
7	Canada	553	0.088 (17)	224 (7)	329 (8)
8	South Korea	508	0.119 (8)	164 (9)	344 (7)
9	Italy	442	0.074 (21)	182 (8)	442 (10)
10	Spain	410	0.083 (18)	128 (12)	282 (9)
11	Netherlands	385	0.110 (11)	164 (9)	221 (11)
12	Switzerland	346	0.136 (3)	143 (11)	203 (12)
13	Australia	301	0.069 (23)	109 (14)	192 (14)
14	India	300	0.069 (24)	105 (15)	195 (13)
15	Sweden	279	0.113 (9)	110 (13)	169 (15)
16	Denmark	208	0.149 (1)	83 (16)	125 (18)
17	Israel	195	0.129 (4)	57 (19)	138 (17)
18	Taiwan	184	0.070 (22)	41 (20)	143 (16)
19	Scotland	158	0.106 (12)	35 (22)	123 (19)
20	Finland	156	0.126 (6)	75 (17)	81 (22)
21	Belgium	147	0.076 (20)	64 (18)	83 (21)
22	Austria	128	0.096 (14)	40 (21)	88 (20)
23	Singapore	109	0.112 (10)	31 (27)	78 (23)
24	Russia	102	0.028 (27)	35 (22)	67 (24)
25	Brazil	99	0.031 (26)	38 (22)	61 (27)
	South Africa (38)	28	0.033 (25)	12 (36)	16 (41)
	Bangladesh (52)	10	0.096 (15)	3 (49)	7 (53)

China (and to a lesser extent Singapore, South Korea, Taiwan and Austria) moved up in the rankings when comparing the second period to the first one. Among the top countries, Japan lost two positions in the rankings. The ranking according to the percentage of articles devoted to Synthetic Biology shows that, on the one hand, Denmark, Israel, Finland and Singapore have a high percentage of articles on SB, and even Bangladesh ranks 15th. On the other hand China, although ranking second in the second period, is only 16th in the ranking per percentage devoted to SB, illustrating the fact that China has many other priorities. Also, Canada, France, Italy and Spain have other priorities. Compared with the results of Oldham, Hall & Burton (2012) we notice several differences: the UK is second in their core group, Switzerland 5th, Spain 6th, Japan 8th and China 10th. However in the citing articles group China becomes 4th.

We divided by continent and obtained the results shown in Table 4. Note that because whole counting has been used, the sum (17,648) is more than the real total (13,836), hence we show results as percentages of the total (and even then results should be interpreted as approximations). Russia is considered to be a part of Europe, not for geographical reasons (then it would be part of Asia) but because most research is performed in the European part of Russia. North America consists of Canada and the USA, while the other countries of the Americas are referred to as Latin America. Compared with the total output of the world, Africa’s share is smaller than 1%. Clearly, Europe and North America keep each other in balance while Asia is the upcoming third.

**Table 4:** Shares per continent.

Continent	Share (in %)
Europe	37
North America	37
Asia	22
Latin America	2
Oceania	2

Most active organizations are shown in Table 5. This list is clearly dominated by American universities, but since the day we collected the data CAS has overthrown MIT as the most-active organization. Yet, this list has no clear top university or small group of top organizations but numbers decrease slowly. We further note that the first company in this list is Genentech Inc. on rank 185 with 27 articles. This seems to indicate that, although synthetic biology can be considered an applied field it is not yet a field which is ripe for large scale commercialization.

**Table 5:** Most-active organizations.

Organization	# Articles
Massachusetts Institute of Technology (USA)	244
Chinese Academy of Science (P.R. China)	242
Harvard University (USA)	242
Caltech (USA)	228
Stanford University (USA)	221
University of Tokyo (JPN)	203
University of California Berkeley (USA)	197
Duke University (USA)	148
University of Washington (USA)	147
University of Illinois (USA)	138

Again Oldham, Hall & Burton (2012) obtain different results. Their list of most-active organizations consists of the University of California Berkeley, the Swiss Federal Institute of Technology (ETH), Harvard and MIT. We found 121 articles for ETH. Clearly, as already shown on country level, China and Japan are underrepresented in their investigation, while, moreover, our results are more recent.

Delving somewhat deeper into this, we also performed a search for patents in the Derwent Innovations Index (DII) using a similar search query as in the WoS. Contrary to article publishing institutions, patent assignees are mostly Japanese and Korean (see Table 6). However, the numbers of assigned patents are of an order of magnitude less than numbers of publications, confirming the observation that the field is not yet ripe for large-scale commercialization. No Chinese company belongs to this list.

**Table 6:** Most-active assignees (from the DII search).

Assignee name	# Patents
Cellfree SCI KK (= CO LTD) (JP)	25
Macrogen Co Ltd (Korea)	23
Dokuritsu Gyosei Hojin Rikagaku Kenkyush (JP)	17
Shimadzu Corp (JP)	17
Toyoboseki KK (JP)	17
Massachusetts Institute of Technology (MIT) (USA)	12
NEC Electronics Corp (JP)	12
University of Louisiana State University and Agricultural & Mechanical College (USA)	10
Riken KK (JP)	10
University of California (USA)	9

The multidisciplinary aspects of SB are clearly shown by the WoS categories involved in its research. Table 7 shows the top-10 categories which together cover about 63% of all articles. However in total 173 WoS categories were involved. As many journals belong to more than one category, the ten categories shown in Table 7 already add up to more than 100%. Also, Oldham, Hall & Burton (2012) have Biochemistry & Molecular Biology as leading subject category (core and citing articles), followed by Chemistry (for the citing articles group) and Biotechnology & Applied Microbiology (second in the core). We observe that Chemistry and Goldman’s (2014) core category Computer Science are not included in our list. The reason is that we used Web of Science categories, while Oldham, Hall & Burton (2012) and Goldman probably used so-called research areas (but write that they use subject categories). Computer Science as a research area consists of several Web of Science categories such as: *Computer Science Interdisciplinary Applications*; *Computer Science Hardware Architecture* and *Computer Science Theory & Methods*. A similar observation holds for *Chemistry*. Moreover, a different approach was used: we and Oldham, Hall & Burton (2012) counted articles, while Goldman applied network centrality indicators. Finally, we used a more inclusive search query.

**Table 7:** WoS categories most involved in SB research.

WoS categories	% of all articles
Biochemistry & Molecular Biology	31.9
Biotechnology & Applied Microbiology	21.7
Multidisciplinary Sciences	9.1
Biochemical Research Methods	8.0
Mathematical & Computational Biology	6.7
Cell Biology	6.6
Biophysics	6.1
Chemistry Multidisciplinary	5.3
Genetics & Heredity	5.1
Plant Sciences	4.1

Using the five main research domains of the Web of Science, we obtain the following percentages per domain: see Table 8.

**Table 8:** Distribution per large domain.

Research domain	Percentage
Life sciences & biomedicine	74.37
Technology	14.89
Physical sciences	9.94
Social sciences	0.57
Arts & humanities	0.24

As MEDLINE covers more journals in medicine and the life sciences than the WoS, be it that more journals are not peer-reviewed (Hu, 2005), we derived a list of journals from this database publishing the most articles within the field of synthetic biology (see Table 9).

**Table 9:** Twenty journals publishing the most articles on synthetic biology (period 2000–2013); data from MEDLINE/WoK.

Source	# records
PLoS ONE	1,202
Proceedings of the National Academy of Sciences of the United States of America	716
The Journal of Biological Chemistry	562
Nucleic Acids Research	532
Methods in Molecular Biology	449
Biotechnology and Bioengineering	410
Applied Microbiology and Biotechnology	409
Journal of Biotechnology	400
Journal of Molecular Biology	399
Bioinformatics Oxford England	345
Protein Engineering Design Selection Peds	333
Metabolic Engineering	325
BMC Systems Biology	313
BMC Bioinformatics	308
BMC Genomics	301
Biochemistry	294
Biochemical and Biophysical Research Communications	288
Applied and Environmental Microbiology	266
Journal of the American Chemical Society	259
Current Opinion in Biotechnology	259

Research in synthetic biology is often supported by grants from large funding bodies. The WoS yields a list of 8,455 names, although there are many funds occurring under several names. Table 10 shows the most-important ones: NIH USA has more than 1,000 supported articles, while the other ones have at least 200 supported articles each.

**Table 10:** Most important funding organizations.

National Institutes of Health (NIH)—USA
National Science Foundation (NSF)—USA
National (Natural) Science Foundation China
European Union (EU)/European Commission (EC)
Deutsche Forschungsgemeinschaft (DFG)

Oldham, Hall & Burton’s (2012) list of funding institutes is dominated by the National Institutes of Health (NIH), the National Science Foundation (US) and the European Programs. Again, China’s research (funded by NSFC) is underrepresented in their study.

Doing better than evolution has a touch of “playing God” and certainly entails moral obligations and ethical problems, see e.g., Renn & Roco (2006) for a discussion of similar problems in the field of nanotechnology. Adding the topic terms “ethic*” OR “moral*” to the main query led to 54 articles. The largest group (17) is in the WoS Category Ethics, followed by Social Sciences Biomedical (12). Six articles are published in *Environmental Values* and five in *Bioethics*. More than half were published in the latest two years.

### Growth in the number of articles on synthetic biology

**Figure 2:**
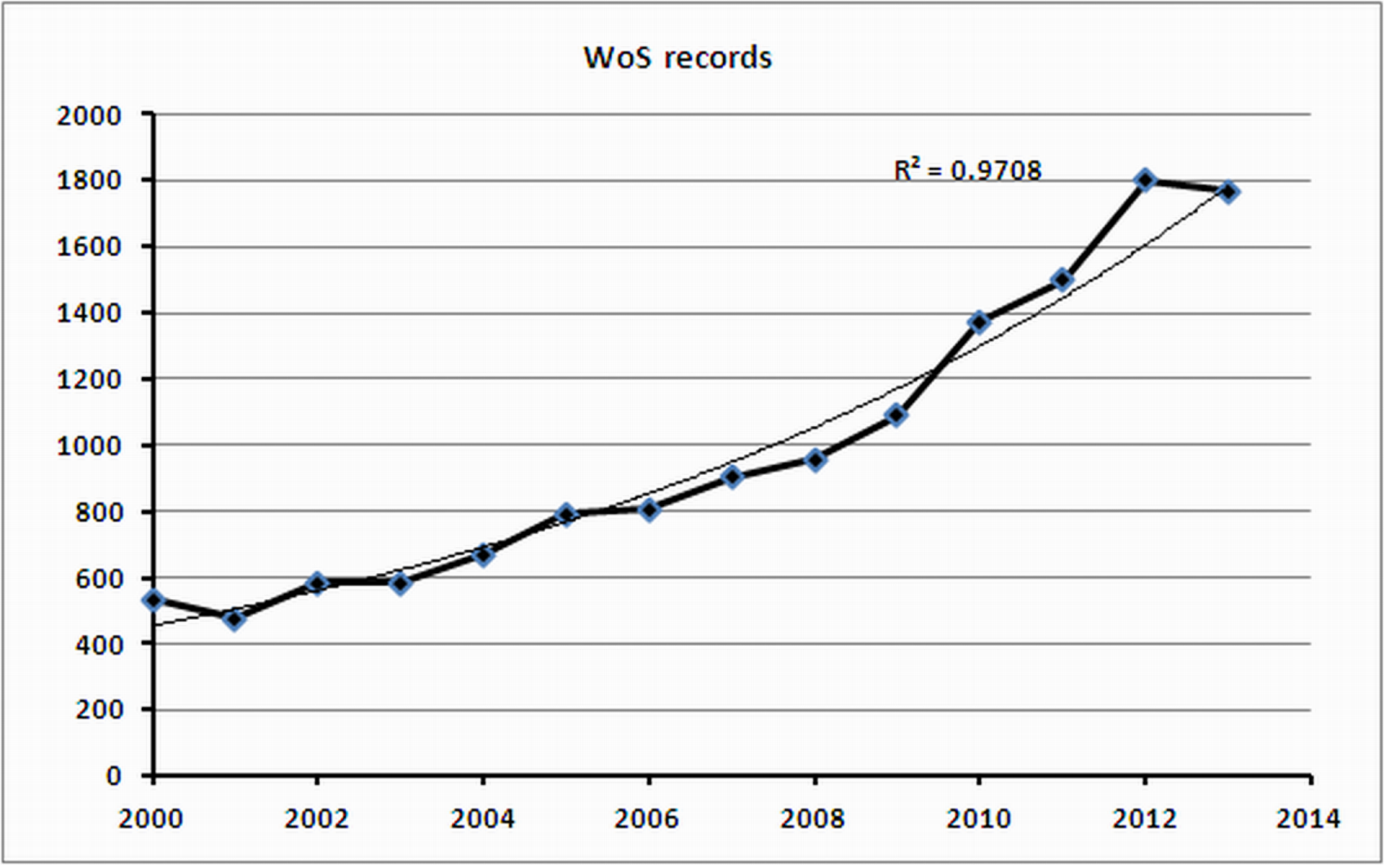
Growth in the number of published articles (WoS data).

The yearly growth curve based on the WoS query is shown in Fig. 2. This curve can best be described as exponential growth. Giving the year 2000 the *x*-value 0 (and hence 2013 the *x*-value 13) a best-fitting curve is given by *y* = 454.3 *e*^0.105*x*^(*R*^2^ = 0.97), where *y* denotes the yearly number of published articles on synthetic biology.

**Figure 3:**
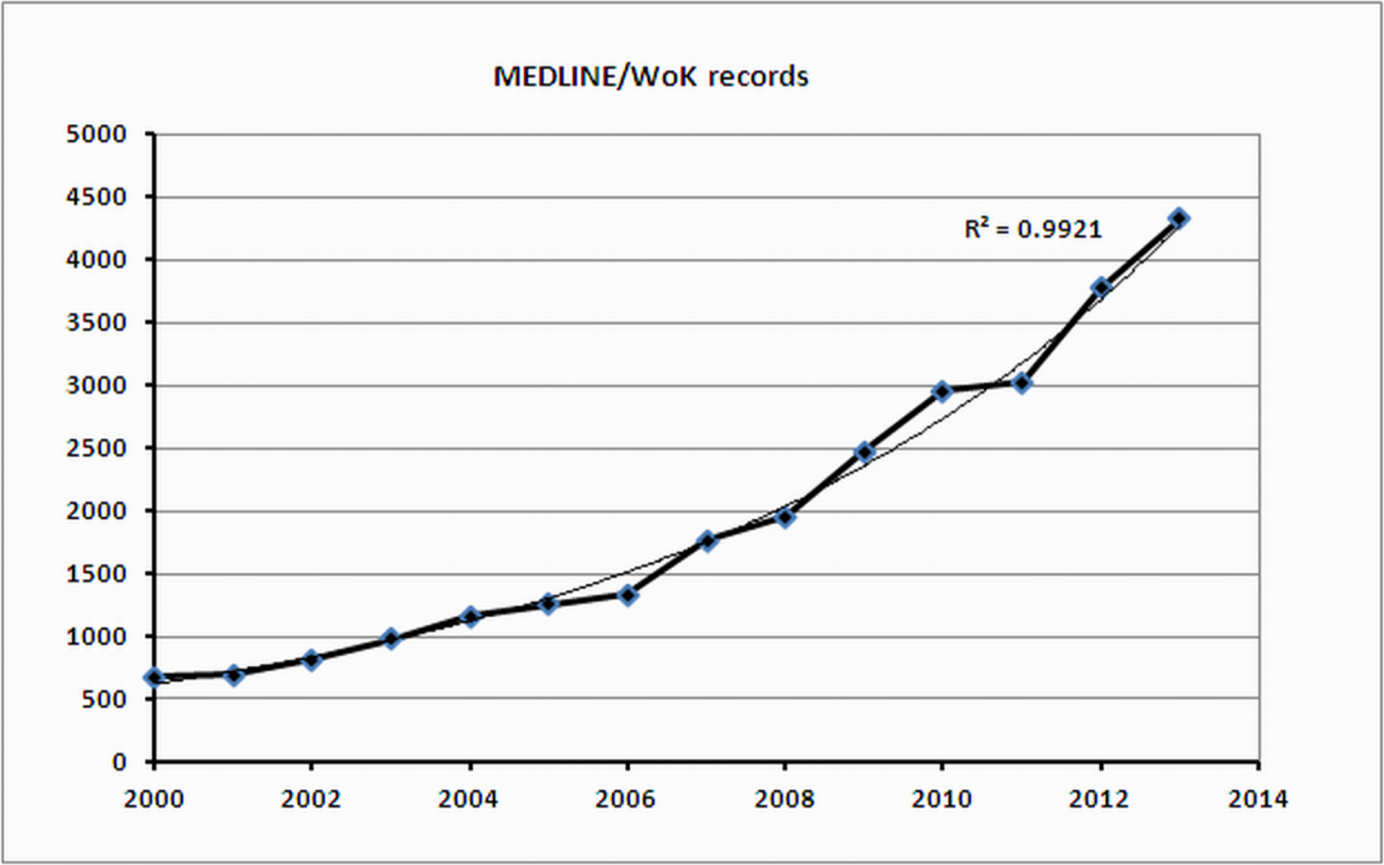
Growth in the number of published articles (MEDLINE data).

This analysis provides an opportunity to compare WoS data with MEDLINE data. Figure 3 shows the growth in number of published articles according to MEDLINE/WoK. Also these data lead to an exponential growth with a best-fitting curve given by *y* = 535.1 *e*^0.148*x*^(*R*^2^ = 0.99). Although our MEDLINE search retrieved considerably more records than the WoS search, the corresponding growth curves show a similar trend.

### Distribution of topic keywords, year-based h-indices

We found a total of 22,253 keywords in the retrieved WoS records. Keywords-PLUS were not included as they are in most cases too general, i.e., not specific for the field of synthetic biology. However, the majority of the keywords (16,905 terms or 76%) occurred just once, reflecting the broadness of the field as well as the fact that, being in an emerging stage, terminology has not yet settled. Remarkably, the term “synthetic biology” (and related terms) occurred just 28 times (in the period of 2000–2013) proving that we had to look into the field’s “world” rather than just considering the “word.” Focusing on major topics, we brought keywords and related forms together under one name. In this way we obtained 35 highly frequent topic-related keywords each occurring at least 100 times. We removed general topics such as cell, enzyme, genetic, gene, protein, *Escherichia coli* and their related terms, leading to 28 keywords representing the hot, specific topics in the field of synthetic biology. These keywords were analyzed using a recently introduced approach based on year-based h-indices (Mahbuba & Rousseau, 2013).

We recall the following definitions from Hu & Rousseau (2014a) and Hu & Rousseau (2014b). Consider a given topic term T and assume that years (here restricted to the period 2000–2013) are ranked according to the number of articles published dealing with this topic. Then this topic’s year-based h-index is equal to *t* if *t* is the highest rank such that in the first *t* years *t* articles were published dealing with this topic (because of the period used, this h-index can at most be equal to 14). Let *Z* and *Y* be the latest and the oldest years included in the topic’s h-core, then the period [*Y*, *Z*] is called the core interval. If *Z*−*Y* + 1 = *t* then there is no gap in the core. The core gap is defined as *Z*−*Y* + 1−*t*, or informally: the number of missing years in the core. Finally, the relative core gap for topic *T* is defined as: (core gap/*t*). In Hu & Rousseau (2014a) and Hu & Rousseau (2014b) we have shown how using these notions may provide an easy-to-use overview of a field concretely: molecular research in nervous system diseases. Table 11 shows the results of the analysis of the SB set, based on year-based h-indices.

**Table 11:** Hot topic keywords in the research field of synthetic biology and their year-based activity h-type indices during the period 2000–2013.

Topic keywords	Year-based h-index	Core interval	Top year	Core gap	Relative core gap
Protein engineering	14	2000–2013	2011	0	0
Metabolic engineering	14	2000–2013	2013	0	0
Protein design+^*^	14	2000–2013	2012	0	0
DNA+	11	2002–2013	2013	1	0.09
MicroRNA+	10	2002–2013	2013	2	0.2
Cell-free protein synthesis+	10	2004–2013	2007	0	0
Protein folding+	10	2000–2011	2004	2	0.2
RNA+	9	2003–2013	2012	2	0.22
Mutagenesis+	9	2004–2013	2002	1	0.11
Gene expression+	9	2002–2013	2013	3	0.33
Stability+	9	2004–2013	2013	1	0.11
Fluorescence+	9	2001–2013	2013	4	0.44
Protein stability+	9	2001–2013	2004	4	0.44
Gene regulatory network+	8	2006–2013	2012	0	0
Directed evolution+	8	2005–2013	2012	1	0.125
Nano+	8	2005–2013	2013	1	0.125
Evolution+	8	2003–2013	2013	1	0.125
Systems biology+	8	2004–2013	2012	2	0.25
Microarray+	8	2006–2013	2012	0	0
Sequential circuit+	8	2000–2008	2000	1	0.125
Biocatalysis+	7	2005–2013	2013	2	0.29
Combination+	7	2002–2013	2013	5	0.71
Gene regulation+	7	2005–2013	2013	2	0.29
Self-assembly+	7	2007–2013	2012	0	0
Antibody+	7	2002–2013	2013	5	0.71
Dynamics+	7	2007–2013	2012	0	0
Protein–protein interaction+	7	2006–2013	2013	1	0.14
Genome+	6	2006-2013	2012	2	0.33

**Notes.**

* The symbol “+” indicates a keyword and its related terms.

Clearly, protein engineering, metabolic engineering and protein design are the overall hot topics in synthetic biology. Oldham, Hall & Burton (2012) found the following top terms: synthetic biology, *E. coli* (a term we removed), gene expression, systems biology and metabolic engineering. Table 10 shows that the core interval of most topics extends to the latest year (namely 2013). Moreover, the top year (the year in which the most articles on this topic were published) is often 2012 or 2013, indicating that interest in these topics is still growing. Interest in sequential circuits seems to have passed its peak.

## Conclusion

We believe that the innovative domain of synthetic biology may become a bigger interdisciplinary domain than nanoscience and nanotechnology. Clearly it is one of the battlefields where leading countries fight for the supremacy in science (Joyce, Mazza & Kendall, 2013). In terms of countries and institutes, the USA is still leading the field, but Mainland China is a strong and upcoming second. The term “synthetic biology” hide a *large world* ready to be explored by interdisciplinary research collaborations. We hope that this informetric study brings a new perspective to the study of synthetic biology.
